# Wireless Communication-Based Indoor Localization with Optical Initialization and Sensor Fusion

**DOI:** 10.3390/s26092653

**Published:** 2026-04-24

**Authors:** Marcin Leplawy, Piotr Lipiński, Barbara Morawska, Ewa Korzeniewska

**Affiliations:** Institute of Information Technology, Lodz University of Technology, al. Politechniki 8, 93-590 Lodz, Poland; piotr.lipinski@p.lodz.pl (P.L.); barbara.morawska@p.lodz.pl (B.M.); ewa.korzeniewska@p.lodz.pl (E.K.)

**Keywords:** inertial navigation, indoor localization, optical localization, RSSI, sensor fusion, Wi-Fi localization

## Abstract

**Highlights:**

- We introduce a wireless communication-based indoor localization method combining optical initialization and inertial sensor fusion.
- The method employs a simplified Factor Graph framework for heterogeneous sensor fusion.
- The system requires minimal infrastructure costs for deployment in modern buildings.
- The system is applicable in environments utilizing mobile devices as access tokens.
- We conducted experiments with a robot to obtain a reliable comparison of methods.

**Abstract:**

Indoor localization in GNSS-denied environments remains a significant challenge due to the low sampling frequency and high variability of wireless signal measurements. This paper presents a wireless communication-based indoor localization method that integrates Wi-Fi received signal strength indication (RSSI) measurements with optical initialization and inertial sensor fusion. The proposed approach eliminates the need for labor-intensive fingerprinting and specialized infrastructure by leveraging existing Wi-Fi networks. Optical pose estimation using ArUco markers provides accurate initial position and orientation, enabling alignment between sensor coordinate systems and reducing inertial drift. During tracking, inertial measurements compensate for motion between sparse Wi-Fi observations by virtually translating historical RSSI samples, allowing statistically consistent averaging and improved distance estimation. A simplified factor graph framework is employed to fuse heterogeneous measurements while maintaining computational efficiency suitable for real-time operation on mobile devices. Experimental validation using a robot-based ground-truth reference system demonstrates sub-meter localization accuracy with an average positioning error of approximately 0.40 m. The proposed method provides a low-cost and scalable solution for indoor positioning and navigation applications such as access-controlled environments, exhibitions, and large public venues.

## 1. Introduction

The rapid growth of Internet-connected devices has significantly increased the demand for accurate indoor localization technologies. This demand is particularly evident in environments where Global Navigation Satellite Systems (GNSS) cannot operate reliably, such as indoor or underground spaces. In such scenarios, satellite signals are heavily attenuated by building structures and affected by multipath propagation, which makes GNSS-based positioning unreliable or completely unavailable [1].

Accurate localization in GNSS-denied environments is an important research problem with applications in robotics, indoor navigation, security systems, and asset tracking [2]. Numerous commercial indoor positioning systems have been developed, including InfSoft [3], Zebra [4], Sonitor [5], Sewio [6], and Cisco [7]. Although these systems often provide good localization accuracy, they typically require dedicated infrastructure, specialized hardware, or extensive deployment procedures, which significantly increase system cost and complexity. As a result, an important design challenge in indoor localization systems is achieving acceptable positioning accuracy while minimizing infrastructure requirements [8].

One attractive solution to this problem is the use of existing wireless communication infrastructure, particularly Wi-Fi networks, which are already widely deployed in modern buildings. In such systems, localization is typically based on Received Signal Strength Indication (RSSI) measurements. However, RSSI-based localization is often associated with limited accuracy due to signal variability, multipath propagation, and environmental dynamics [9]. In contrast to many existing studies that primarily attribute localization errors to high RSSI variance [10,11], our work emphasizes another fundamental limitation of Wi-Fi-based localization systems: the low sampling frequency of RSSI measurements.

Experimental observations indicate that RSSI measurement errors tend to follow an approximately Gaussian distribution when a sufficient number of samples is collected. However, under non-line-of-sight (NLOS) conditions the distribution may become multimodal due to reflections and environmental interference [12]. As a result, RSSI-based localization can achieve relatively good accuracy for stationary objects, but its performance deteriorates significantly when the tracked object is in motion [13,14].

In general, localization systems must provide measurements at a sufficiently high frequency to estimate object position continuously over time [15]. When the measurement frequency is too low relative to the velocity of the moving object, large positional changes may occur between consecutive measurements, leading to substantial localization errors. This situation is common in Wi-Fi-based systems. For example, typical RSSI sampling frequencies in mobile devices are approximately 0.5 Hz, while localization errors in controlled environments often exceed 3 m [16]. Even for slowly moving users, such as pedestrians walking at approximately 6 km/h, the available measurement frequency and accuracy are often insufficient for reliable localization [13,17].

A common strategy for improving localization accuracy in such scenarios is the fusion of multiple measurement systems. In many existing approaches, motion models are used to estimate the object’s movement between individual measurements. However, this approach introduces additional challenges, particularly because different sensing modalities often operate in distinct coordinate systems. Accurate alignment of these coordinate frames is therefore necessary to effectively combine heterogeneous measurements.

Considering these challenges, the present study focuses on two key aspects that are essential for improving localization accuracy in low-frequency measurement environments:

1. Determining the relative positioning of coordinate systems in which localization measurements are performed.
2. Developing localization methods capable of operating under conditions where the measurement frequency is significantly lower than the maximum velocity of the moving object.

In this work, we consider practical scenarios in which users equipped with smartphones move through GNSS-denied environments such as stadiums, concert venues, exhibition halls, or trade fairs [18]. In such environments, access control is typically implemented through electronic tickets displayed on mobile devices and verified using QR codes or barcodes [19].

Our proposed system extends this concept by combining QR-based ticket verification with optical localization using Augmented Reality University of Cordoba (ArUco) markers. During the ticket validation process, the optical subsystem determines the precise six-degree-of-freedom (6D) pose of the mobile device. This provides centimeter-level initialization of the device position and orientation, which can then be used as the starting point for indoor localization within a two-dimensional (2D) plane. Furthermore, the orientation parameters obtained from the ArUco marker allow calibration of the mobile device’s inertial sensors, including the magnetometer, gyroscope, and accelerometer.

By accurately determining the initial pose of the device, the proposed system enables the alignment of coordinate systems used by different sensing modalities. This alignment allows high-frequency inertial measurements obtained from the mobile device to be effectively fused with low-frequency Wi-Fi RSSI measurements. As a result, the proposed approach improves localization accuracy and robustness while maintaining low deployment costs by leveraging existing Wi-Fi infrastructure. Unlike fingerprinting-based systems, the proposed approach explicitly exploits motion information to compensate sparse RSSI measurements, enabling accurate localization with minimal infrastructure requirements. The remainder of this paper is organized as follows. Section 2 reviews related work on indoor localization. Section 3 formally defines the localization problem. Section 4 describes the proposed method in detail. Section 5 presents the experimental setup. Section 6 reports and discusses the experimental results. Section 8 concludes the paper and outlines directions for future work.

## 2. Related Work

Various methods have been proposed to improve RSSI-based localization accuracy. Initial instances of positioning systems employing fingerprinting include the RADAR system [20], which employs empirical measurements with signal propagation modeling followed by the *K*-Nearest Neighbor method and Horus [21] systems which employs a probabilistic paradigm, accounting for the statistical attributes of RSSI. These methods have been improved by applying various mathematical models, such as the Lognormal distribution [21], Weibull function [22], Gaussian distribution [23], and the double peak Gaussian distribution [24]. Machine learning (ML) methods have also been widely applied to indoor navigation, as demonstrated in [13,14,25,26], due to their robustness to signal variability.

Among these algorithms are the Naive Bayes classifier [27], Support Vector Machine [28], Random Forest [29], and Neural Network [30]. Other approaches include unsupervised learning [31], Hyperbolic Location Fingerprinting [32], the Signal Strength Difference method [33], Online Random Forest [34], multiple classifiers [35], RSSI Quality Evaluation Model [36], Deep Neural Networks followed by Convolutional Neural Network, and Dempster–Shafer theory of subjective probability [37], Extreme Gradient Boosting [38], median-based clusterization [39], and fingerprints augmentation [40]. However, all these methods require reference signal fingerprinting, which makes deployment across different environments time-consuming and costly. Another approach involves angle-of-arrival measurements and advanced Wi-Fi sensing techniques [41] but requires dedicated specialized hardware, the same as the use of Time of Flight for example in publication [42] or multichannel approach [43]. These algorithms often produce better results than our algorithm, but they require specialized hardware to deploy.

In contrast to the reviewed approaches, the key contributions of our system are as follows:

- We propose a novel hybrid localization architecture combining optical initialization (via ArUco), inertial sensor tracking, and Wi-Fi RSSI-based updates in a unified framework.
- Our method does not rely on fingerprinting, calibration databases, or specialized hardware, making it low-cost and scalable.
- We introduce a fusion algorithm based on the Factor Graph that effectively compensates for sensor drift and Wi-Fi latency, achieving sub-meter accuracy in real-world conditions.

The novelty of the proposed approach lies in motion-compensated averaging of Wi-Fi RSSI measurements combined with optical initialization using ArUco markers, which allows reliable localization despite the low sampling rate of Wi-Fi signals. This approach differentiates our work from prior solutions that either require dense infrastructure (e.g., Ultrawideband or Bluetooth Low Energy) or involve high calibration and computational overhead, such as fingerprinting or machine learning-based methods. Compared to these technologies, the proposed system offers a favorable trade-off between cost and performance. While UWB provides high localization accuracy (typically within 10–30 cm), it requires specialized infrastructure and dedicated anchors, leading to high deployment costs. Bluetooth Low Energy, although low-cost, suffers from significantly shorter range than Wi-Fi, making it less suitable for large or open indoor environments. The proposed system, by leveraging existing Wi-Fi infrastructure and optical initialization, achieves competitive sub-meter accuracy with minimal cost and operational complexity, making it an effective and scalable solution for dynamic access control scenarios in GNSS-denied environments.

## 3. Problem Definition

More formally, the problem of object localization of a tag over time within a two-dimensional plane (x,y) can be defined as the search for a localization function *L*, given by:

(1) $$x\left(t\right)=\left(x\left(t\right),y\left(t\right)\right)=L\left(x_{0},D\left(t\right),a\left(t\right),\omega \left(t\right),M\left(t\right)\right)$$

where:

- $x_{0}$—initial position in the (x,y) plane,
- $D\left(t\right)=[D_{1}\left(t\right),D_{2}\left(t\right),\ldots ,D_{k}\left(t\right)]$—distances between the tag and Wi-Fi access points with known positions,
- a(t)—three-dimensional acceleration measured by the accelerometer,
- ω(t)—three-dimensional angular velocity measured by the gyroscope,
- M(t)—three-dimensional magnetic field measured by the magnetometer.

The objective of the localization function is to minimize the mean squared error (MSE) between the estimated position x(t) and the reference position $x_{\mathrm{ref}}\left(t\right)$ over the measurement period *N*, defined as:

(2) $$E=\frac{1}{N+1}\sum_{t=0}^{N}{∥x\left(t\right)-x_{\mathrm{ref}}\left(t\right)∥}^{2}.$$

where:

- *N*—measurement period,
- $x_{\mathrm{ref}}\left(t\right)=\left(x_{\mathrm{ref}}\left(t\right),y_{\mathrm{ref}}\left(t\right)\right)$—the reference position of the mobile device on the (x,y) plane.

Our objective is to minimize the mean squared error across all localization measurements of the tag over the measurement period. In principle, the optimal localization of the tag can be obtained by considering all historical measurements together with the initial position. However, this approach is suboptimal due to its high memory and computational requirements. In practice, the number of historical samples should be adjusted according to the characteristics of the localization system, sensor properties, and the required accuracy.

In the context of mobile device localization using Wi-Fi signal strength measurements, the conventional framework given in Equation (1) can be adapted by incorporating knowledge of the system characteristics. The objective is to derive a mathematical formulation that maps Wi-Fi, accelerometer, gyroscope, and magnetometer measurements to a position estimate that minimizes the error defined in Equation (2).

## 4. Proposed Method

**Figure 1:**
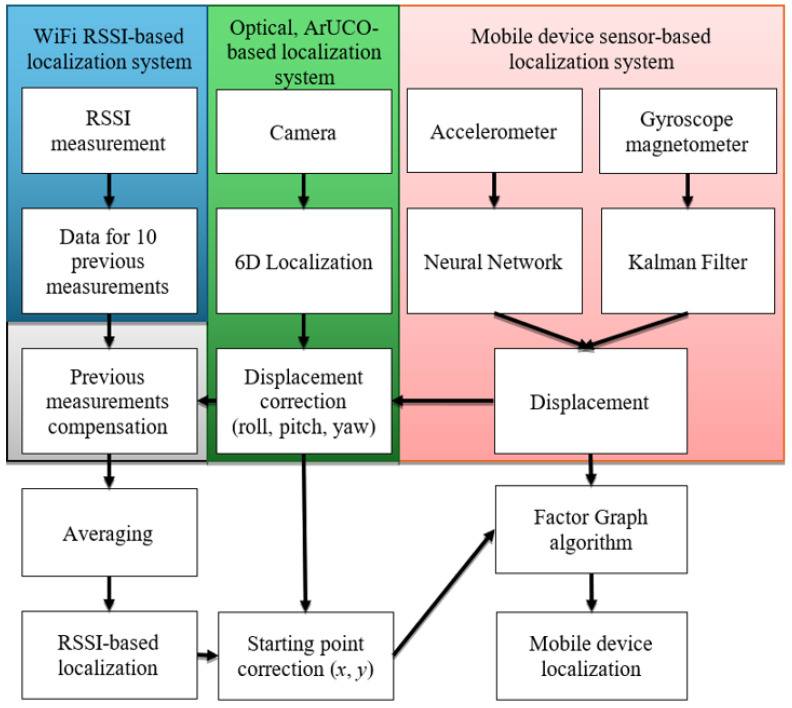
Dynamic, Hybrid, Optical–Wi-Fi Mobile Device Localization System diagram.

The experiments described in this paper were conducted using a robotic platform in order to obtain precise and repeatable ground-truth reference data. However, the system is designed for deployment on handheld mobile devices carried by human users. The robotic platform serves solely as a controlled test vehicle; the sensing modalities, algorithms, and fusion framework are identical to those intended for the human-carried scenario. The key factors determining the performance of *L* are the accuracy, reliability, and sampling frequency of each sensor modality. The proposed methodology addresses the challenge of ascertaining the position of a mobile device amidst conditions marked by insufficient Wi-Fi RSSI sampling frequency relative to the object’s velocity. In such scenarios, the object may traverse a considerable distance between localization measurements, potentially leading to significant measurement inaccuracies, particularly in instances of high variance, a characteristic often encountered in Wi-Fi RSSI signals. Such an approach is also valid when the object is stationary, as it computes the average RSSI signal value over relatively long period of time. Our experimental results demonstrate that the accuracy of the RSSI-based measurement improves with the duration of data capture at a fixed location. Over time, the distribution of Wi-Fi RSSI values approximates a Gaussian distribution. As a result, utilizing the mean RSSI value as a location estimator remains statistically valid. Specifically, for non-moving objects—particularly in real-world environments—averaging the RSSI values enhances localization accuracy by mitigating the effects of temporal environmental fluctuations. Therefore, as the stationary duration increases, RSSI-based accuracy improves, allowing for more samples to be averaged without the introduction of motion-induced variance. Mobile devices are equipped with cost-effective onboard sensors operating at substantially higher frequencies compared to Wi-Fi RSSI. However, these sensors are imprecise and accumulate errors over time, as they are difficult to calibrate in real-life scenarios. Additional challenges involve integrating data from these disparate systems, each utilizing distinct reference frameworks, and determining the initial point of the measurement trajectory. The current implementation does not explicitly account for acceleration bursts, irregular walking speed, or phone shaking, which are common in real-world usage. Additionally, changes in device orientation (e.g., holding the phone at varying angles) may affect both RSSI measurements and inertial sensor readings. These factors will be investigated in future work. To address these challenges, the authors devised the localization system. The architectural layout of the system is depicted in Figure 1, comprising three primary components:

1. A mobile device sensor-based localization system.
2. An optical, ArUco-based localization system.
3. A Wi-Fi RSSI-based localization system.

### 4.1. Mobile Device Sensor-Based Localization

The first system component, highlighted in red within Figure 1, harnesses data from the accelerometer, gyroscope, and magnetometer inherent in contemporary mobile devices. The gyroscope and magnetometer ascertain the heading of the mobile device. In our approach, we employ the Kalman filter for data fusion. The IMU-based displacement is propagated according to:

(3) $$x_{t+1}=x_{t}+\Delta x_{t}+\eta_{t},\;\eta_{t}∼N\left(0,\sigma_{\mathrm{IMU}}^{2}I\right)$$

where $\Delta x_{t}$ is the displacement estimated from the neural network velocity output and heading from the Kalman filter, and $\eta_{t}$ is zero-mean Gaussian noise with variance $\sigma_{\mathrm{IMU}}^{2}$.

The accelerometer is utilized for measuring the distance traveled. However, based on our empirical observations and findings presented in subsequent references [44,45,46] distance calculations relying on the double integration of accelerometer measurements result in notable errors. This is attributed to the substantial bias values inherent in the accelerometers of mobile devices, posing challenges for effective mitigation. As a result, we adopt an approach wherein we initially compute the acceleration amplitude for each sample. Subsequently, we transform the last 256 samples into the Fourier domain, as determined by the following relation:

(4) $$A_{l}=\sum_{n=0}^{N-1}a_{n}\omega_{N}^{-ln},\;0\leq l\leq N-1,$$

where:

(5) $$\omega_{N}=e^{j\frac{2\pi }{N}}$$

- *j*—the imaginary unit,
- *l*—the harmonic number,
- *n*—the signal sample number,
- $a_{n}$—the signal sample value,
- *N*—the number of samples (256 samples were used in this work).

Equations (3) and (4) represent the standard Discrete Fourier Transform definition [47].

Subsequently, we compute the absolute value of the first 128 samples of the Fourier transform output, denoted as $|A_{l}|$, and employ them as inputs into an artificial neural network. This approach was chosen because the mobile phone was mounted on a robot, which provided consistent and repeatable motion for training data collection. Alternative approaches may be more suitable under different testing conditions, as outlined in, for instance [44,45,46].

Initially, we train the neural network by inputting accelerometer sequences corresponding to various velocities. We employ a feedforward neural network comprising 128 inputs corresponding to the magnitude spectrum of the DFT, three hidden layers with 256, 128, and 64 neurons respectively, ReLU activation functions, and a single linear output neuron regressing the scalar velocity magnitude in the (x,y) plane. plane. The network was trained using the Adam optimizer with a learning rate of 0.001 and mean squared error loss over 600 training sequences (480 training, 60 validation, 60 test) collected at robot velocities of 0.2, 0.4, and 0.6 m/s. Training was performed offline prior to deployment; no online retraining is performed during localization. The pre-trained network estimates the velocity in the (x,y) plane. Combined with the heading from the Kalman filter (magnetometer and gyroscope), this gives the device displacement $\Delta x_{t}$ at each time step. To partially address device heterogeneity, the training sequences were collected on the same device used in the experiments (Xiaomi Redmi Note 10). Cross-device generalization of the velocity estimator will be investigated in future work. Formally, let $x_{t}$ denote the device state (position) at time *t*. The factor graph consists of variable nodes $\left\{x_{t}\right\}$ connected by two types of factors:

- Binary (IMU) factors:
  (6) $$f_{\mathrm{IMU}}\left(x_{t},x_{t+1}\right)\propto \exp\left(-\frac{{∥x_{t+1}-x_{t}-\Delta x_{t}∥}^{2}}{2\sigma_{\mathrm{IMU}}^{2}}\right)$$
- Unary (Wi-Fi) factors:
  (7) $$f_{\mathrm{Wi}-\mathrm{Fi}}\left(x_{t}\right)\propto \exp\left(-\frac{{\left(d_{\mathrm{est},t}-d_{\mathrm{meas},t}\right)}^{2}}{2\sigma_{\mathrm{Wi}-\mathrm{Fi}}^{2}}\right)$$

where $\Delta x_{t}$ is the displacement estimated from inertial data, $d_{\mathrm{est},t}=∥x_{t}-x_{\mathrm{AP}}∥$ is the Euclidean distance between the state $x_{t}$ and the access point position $x_{\mathrm{AP}}$, and $\sigma_{\mathrm{IMU}}^{2}$ and $\sigma_{\mathrm{Wi}-\mathrm{Fi}}^{2}$ are the respective noise variances.

The MAP estimate is obtained by minimizing the negative log-likelihood over all factors.

### 4.2. Optical ArUco-Based Localization

**Figure 2:**
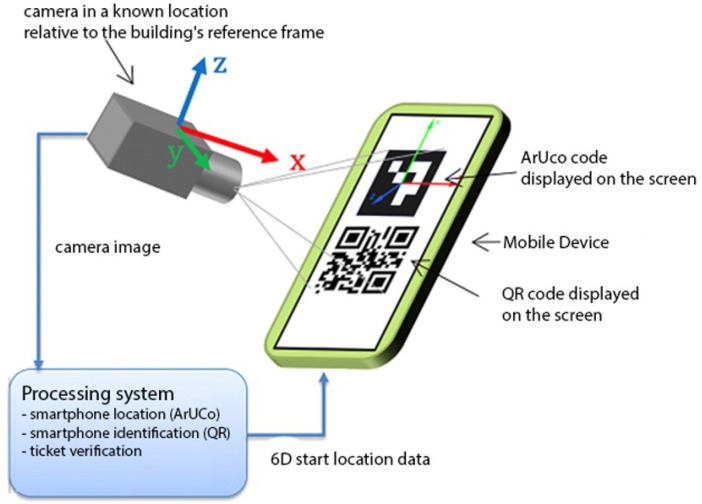
Component of system: optical, ArUco-based localization system.

The second component of the system, referred to as the optical ArUco-based localization subsystem, highlighted in green in Figure 2, utilizes ArUco markers developed in 2014 at the University of Cordoba. ArUco markers represent a type of matrix codes capable of accurately determining their six-dimensional (6D) spatial location, encompassing the three-dimensional position (x, y, z) and rotation around the X, Y, and Z axes (roll, pitch, yaw), with high precision using a camera. Our experiments indicate that the 6D localization error values, obtained with the aforementioned software, employing a 14-megapixel camera and maintaining a 10-cm distance to the displayed marker on a mobile device screen, remain below 0.5 cm for (x, y, z) coordinates and below 0.017 radians for roll, pitch, and yaw orientations. This methodology facilitates the determination of the initial measurement position and the calibration of the magnetometer and gyroscope if the camera localization is known a priori. In cases of orientation discrepancies between the optical and sensor frames, the system prioritizes the orientation obtained from the optical (ArUco-based) localization due to its significantly higher accuracy. Due to the superior precision of the optical system relative to the inertial sensors, the optical frame is designated as the reference, facilitating the correction of sensor biases and misalignments. Subsequently, the localization data needs to be transmitted to the mobile device for further mobile phone localization. As a result, the system must identify the mobile phone. However, ArUco codes, with only 1020 combinations in the original ArUco dictionary, offer insufficient capacity for this purpose. Thus, we opt for QR codes, which possess significantly higher informational capacity, to identify mobile devices. Naturally, the mobile device must be equipped with an application capable of displaying both ArUco and QR codes and communicating with the ArUco-based localization system to obtain the initial position of the mobile device. In practical scenarios, this application can be integrated with a ticketing system, displaying the ticket in the form of a QR code, which is subsequently verified at the entrance to the area where the mobile device is localized.

In edge cases where the ArUco marker or QR code is not detected—e.g., due to unfavorable camera angle, occlusion, or user error—the system still continues localization based on Wi-Fi RSSI and inertial sensor data. However, the localization accuracy is reduced due to the lack of initial calibration. In real-world applications, particularly those involving access control (e.g., stadiums, events, or exhibitions), the absence of a visible marker typically prevents user entry altogether. As such, these scenarios are largely theoretical and do not affect practical system deployment.

### 4.3. Wi-Fi RSSI-Based Localization and Fusion

**Figure 3:**
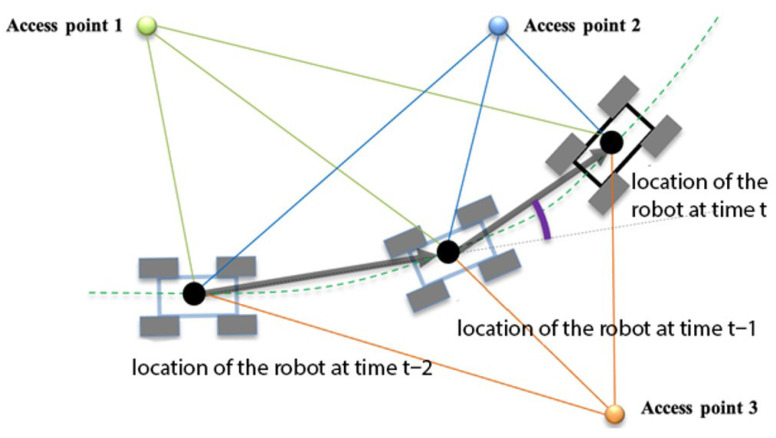
Component of the system: Wi-Fi RSSI-based localization diagram. The colored lines represent distances from the mobile device to individual Wi-Fi access points, while the gray vectors indicate the estimated displacement derived from inertial measurements.

The last component of the system is the Wi-Fi RSSI-based localization system shown in Figure 3. It uses Wi-Fi RSSI to measure the distance between the access points and the mobile device. The receiver’s distance from the transmitter affects the power of the received signal. To precisely estimate this distance, one must apply a propagation model that reflects the environmental conditions in which the measurement takes place. The most commonly used model assumes that the signal power, expressed in decibels, is proportional to the logarithm of the distance. As a result, the distance can be expressed as:

(8) $$d=10^{\left(\frac{P_{\mathrm{tx}}-{\mathrm{CL}}_{\mathrm{tx}}+{\mathrm{AG}}_{\mathrm{tx}}+{\mathrm{AG}}_{\mathrm{rx}}-{\mathrm{CL}}_{\mathrm{rx}}-P_{\mathrm{rx}}-\mathrm{FM}-K-20\log_{10}\left(f\right)}{20}\right)},$$

where:

- *d*—distance between the receiver and the transmitter,
- *K*—coefficient equal to 27.55, applied when the frequency is expressed in MHz and the distance in meters; this value may be adjusted depending on operating conditions,
- $P_{\mathrm{tx}}$—transmitted signal power,
- ${\mathrm{CL}}_{\mathrm{tx}},{\mathrm{CL}}_{\mathrm{rx}}$—cable losses; if no cables are used, the losses are 0 dB,
- ${\mathrm{AG}}_{\mathrm{tx}},{\mathrm{AG}}_{\mathrm{rx}}$—antenna gains,
- $P_{\mathrm{rx}}$—received signal power (receiver sensitivity),
- FM—fade margin, defined as the difference between the received signal strength and the minimum level required for correct reception,
- *f*—signal frequency in MHz [15].

The algorithm is illustrated in Figure 3, showing a robot navigating along a path (green dashed line). Figure 3 portrays the robot’s spatial coordinates at three sequential time points (*t*, t−1, t−2), coinciding with the instances of position measurement utilizing Wi-Fi Received Signal Strength Indication (RSSI). The depicted distances from the robot to each of the access points during consecutive time points are represented by color-coded segments: green, blue, and orange, corresponding to Access Points 1, 2, and 3, respectively.

At each of these specified positions, the robot’s spatial coordinates were determined by employing the multilateration algorithm. Concurrently, the robot’s displacement between the temporal instances (*t*, t−1, t−2) is delineated by gray vectors. The vector magnitude is derived from data obtained from the accelerometer and artificial neural network, while the vector angle, denoted in the robot’s coordinate system and marked in purple, is ascertained utilizing the Kalman filter with data sourced from the accelerometer and gyroscope.

The distance measurements obtained via Wi-Fi RSSI exhibit a considerable standard deviation, potentially reaching up to 5 m. Furthermore, the measurement frequency of the Wi-Fi RSSI signal is notably low, approximately 0.5 Hz in mobile devices. Under such circumstances, the dynamic localization based on Wi-Fi RSSI proves to be a challenging task.

In our experimental investigations, we observed that in unobstructed environments, discrete distance measurements and their corresponding localization data exhibit a normal error distribution. This characteristic implies that the mean value effectively approximates the true value. However, when an object moves swiftly relative to the frequency of the measurement system, as is the case in our study, utilizing the mean value becomes impractical due to the object’s varying position during each measurement. Therefore, in our methodology, we employed displacement data from a mobile device sensor-based localization system to account for the object’s movement relative to preceding measurement points. This strategy enables the averaging of compensated measurements, thereby deriving an average from virtually translated previous measurements, which substantially enhances measurement stability. Furthermore, this approach partially compensates for multipath fluctuations as the object traverses.

**Figure 4:**
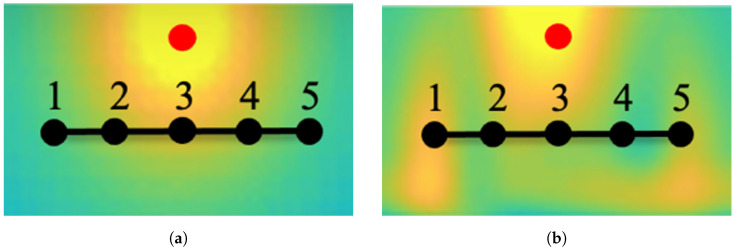
(**a**) Ideal RSSI distribution originating from the access point positioned at the red dot. (**b**) Sample measured RSSI distribution from the access point located at the red dot.

This compensation method is elucidated schematically in Figure 4. Figure 4a illustrates the RSSI distribution according to our model, while Figure 4b depicts real measurements inclusive of signal reflections from surrounding walls, based on [3]. The red dot denotes the access point, while the black line traces the mobile device’s localization across five consecutive measurement points from 1 to 5. Measurements at points 1, 2, 4, and 5 are influenced by signal reflections from walls, resulting in higher RSSI values. Notably, the RSSI values at points 1 and 5 exceed those predicted by our model, while values at points 2 and 4 are lower. By applying averaging to compensated measurements, the resultant distance from the access point proves more accurate than direct measurements at point 5. Note that this simplified explanation serves to elucidate our approach, with comprehensive results presented in subsequent sections.

**Figure 5:**
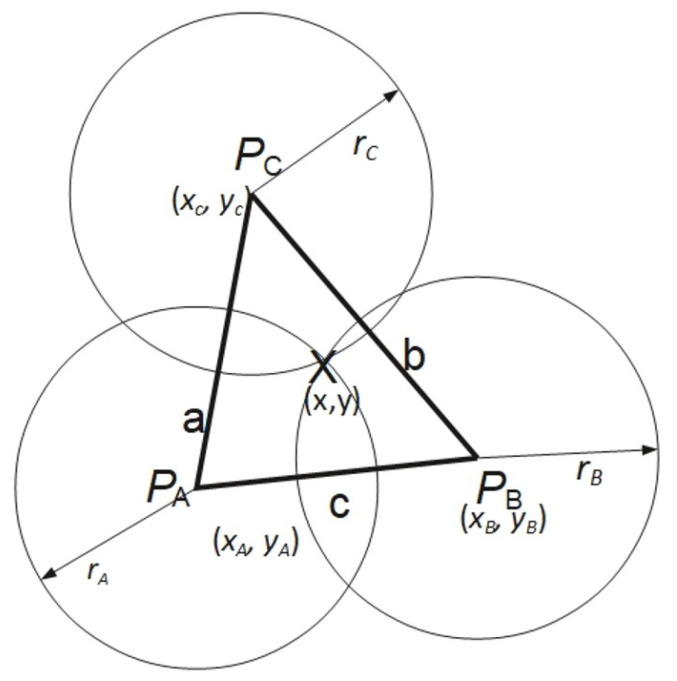
Multilateration algorithm: ideal case.

The distance measurements from multiple Wi-Fi access points are then used to find the mobile device localization using a multilateration algorithm. In the two-dimensional variant of this method, in which the object’s position is determined based on the distances between the tag and at least three access points, as schematically depicted in Figure 5. The anchor locations are described in the figure as points $P_{1}\left(x_{1},y_{1}\right)$, $P_{2}\left(x_{2},y_{2}\right)$, and $P_{3}\left(x_{3},y_{3}\right)$. Each of the three circles centered at points $P_{1}$, $P_{2}$, and $P_{3}$ has respective radii $r_{1}$, $r_{2}$, and $r_{3}$ determined based on distance determination algorithms described previously in this section. The figure illustrates the ideal scenario where all circles intersect at a single point. In practical measurements, achieving a common intersection point for three circles is difficult to accomplish. Hence, it is necessary to approximate the position. The approximate location can be obtained using various methods depending on the measured distances between the tag and anchors, as well as the number of anchors. In situations where the number of anchors exceeds three, the tag’s position can be determined as a function that minimizes the sum of squared errors, as described by Formula (9) [48].

(9) $$\epsilon =\sum_{i=1}^{N}{\left(∥X-P_{i}∥-r_{i}\right)}^{2},\;$$

where:

- X(x,y)—the tag localization on a 2D plane,
- $P_{i}\left(x_{i},y_{i}\right)$—localization of the *i*-th access point, where i=A,B,C,…,
- $r_{i}$—radius of the *i*-th circle, where i=A,B,C,….

In cases where the utilization of more than three anchors is not possible, determining the approximate location of point X(x,y) based on Equation (9) is not feasible. The tag’s location can then be determined as the centroid of the triangle formed by the intersection points of pairs of circles centered respectively at PA and PB, PA and PC, and PB and PC. Each pair of circles has two intersection points. To compute the centroid of the triangle, only points lying on the side opposite the third vertex of the triangle with respect to the line determined by the centers of the circles are considered, as illustrated schematically in Figure 5. In the considered scenario, for the pair of circles with centers at points PA and PB, the intersection point lying in the red area is selected; for the pair of circles with centers at points PA and PC, the intersection point lying in the yellow area is chosen, while for the pair of circles with centers at points PB and PC, the point lying in the region marked with blue is selected.

**Figure 6:**
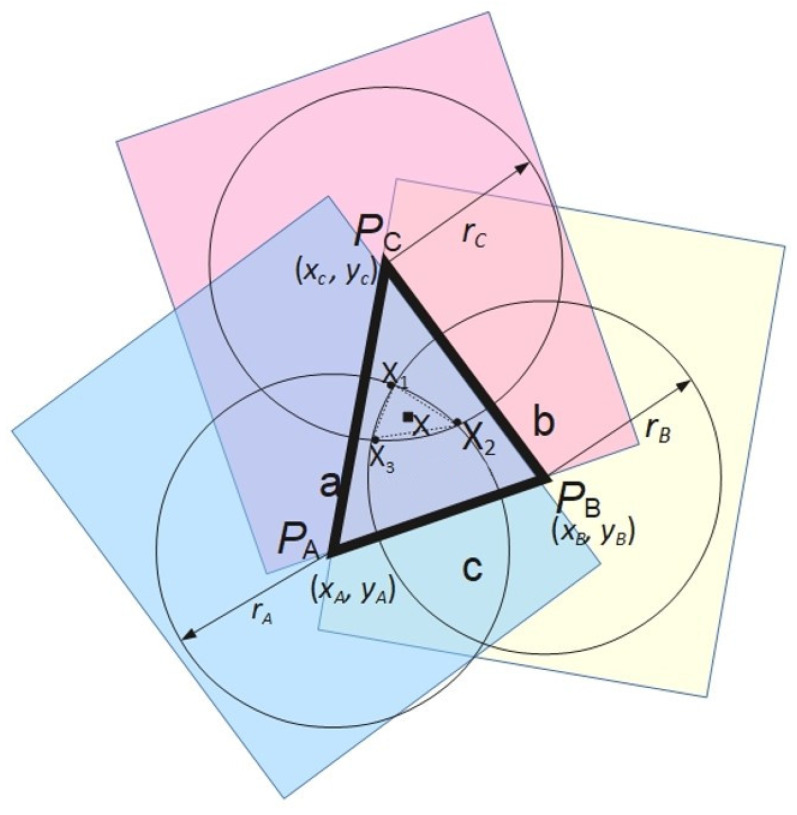
Multilateration algorithm with three access points. Points $P_{A}$, $P_{B}$, and $P_{C}$ denote access point locations, while $X_{A}$, $X_{B}$, and $X_{C}$ represent pairwise circle intersection points used to estimate the final position X(x,y).

The intersection points $X_{A}$, $X_{B}$, $X_{C}$ are shown as black dots in Figure 6, and the centroid X(x,y) (the estimated tag position) as a black square. The coordinates of point *X* in this case are determined by the Equation (10):

(10) $$x=\frac{x_{A}+x_{B}+x_{C}}{3},y=\frac{y_{A}+y_{B}+y_{C}}{3}$$

In cases where a given pair of circles do not intersect and the circles are positioned relative to each other in a manner where one does not lie within the other, the intersection point of the circles is determined based on the ratio of their radii. For the pair of circles PA and PB, the relation is given by Equation (11). The dependencies for the remaining points are analogous.

(11) $$x_{1}=x_{A}+\left(x_{B}-x_{A}\right)\frac{r_{A}}{r_{B}},y_{1}=y_{A}+\left(y_{B}-y_{A}\right)\frac{r_{A}}{r_{B}}$$

On the other hand, in cases where the circles are positioned relative to each other in a manner where one circle lies inside the other, the position of point X1 is determined based on the Equations (12) and (13)

(12) $$x_{1}=\frac{1}{2}\left(x_{A}+x_{B}+\frac{\left(r_{A}+r_{B}\right)\left(x_{A}-x_{B}\right)}{\sqrt{{\left(x_{A}-x_{B}\right)}^{2}+{\left(y_{A}-y_{B}\right)}^{2}}}\right)$$

(13) $$y_{1}=\frac{1}{2}\left(y_{A}+y_{B}+\frac{\left(r_{A}+r_{B}\right)\left(y_{A}-y_{B}\right)}{\sqrt{{\left(x_{A}-x_{B}\right)}^{2}+{\left(y_{A}-y_{B}\right)}^{2}}}\right)$$

Equations (10)–(13) were derived by the authors based on elementary geometric relationships between intersecting and non-intersecting circles.

By employing the three localization systems delineated in this section, it becomes feasible to attain sub-meter localization accuracy for the mobile device, as elucidated in the experimental section by applying the Factor Graph algorithm.

Although the method internally utilizes a simplified version of the Factor Graph-based algorithm, it differs significantly from a standard implementation. In our case, the algorithm does not perform full probabilistic inference with adaptive weight updates typically found in traditional factor graph-based localization. Instead, we apply fixed-weight averaging over adjusted Wi-Fi RSSI measurements, using inertial data to compensate for motion. This simplification is motivated by practical limitations observed during experiments: when applied directly to Wi-Fi-derived inputs, the full Factor Graph formulation often failed to converge or produced unstable results due to low sampling rates and irregular signal behavior. Therefore, while the proposed system adopts a factor graph structure for data fusion, it operates under constrained assumptions, and its behavior differs sufficiently to justify separate performance evaluation.

In the proposed Factor Graph-based implementation, we begin the inference process from a single initial pose provided by the optical localization subsystem. Due to the significantly higher accuracy of the optical method—typically two orders of magnitude better than RSSI-based Wi-Fi localization—the initial measurement is considered reliable and assigned a high confidence weight. As a result, the factor graph is constructed with a chain-like structure starting from this trusted pose, and additional measurements are incrementally integrated using Gaussian noise models specific to each sensor modality. While the general structure of the factor graph algorithm is adopted from standard probabilistic inference frameworks [49], we simplify the weight assignment by assuming fixed high certainty for the optical reference and model-based uncertainty for subsequent RSSI-derived inputs.

From a computational perspective, the most time-consuming component is the neural network responsible for estimating movement velocity from accelerometer data. However, the inference time remains significantly shorter than the Wi-Fi RSSI sampling interval (typically around 2 s), and therefore does not affect real-time operation. Other components, including Kalman filtering and multilateration, complete their computations within a few milliseconds.

Furthermore, since the initial optical localization is highly accurate, the system does not require backward processing of historical Wi-Fi measurements at the start. During the first approximately 20 s, localization proceeds using forward-only processing of incoming Wi-Fi and inertial data. While the algorithm uses a window of 10 Wi-Fi samples (spanning approximately 20 s) to improve stability, this processing is performed incrementally and does not delay position updates.

As a result, the proposed fusion method introduces negligible latency in practical indoor scenarios involving human movement (see Algorithm 1).
**Algorithm 1** Motion-Compensated RSSI Averaging  1:Initialize device pose using optical localization  2:Initialize sliding window *W* for RSSI samples  3:**while** localization system is active **do**  4:      Acquire RSSI measurements from Wi-Fi access points  5:      Estimate device displacement (Δx,Δy) using inertial sensors  6:      Append RSSI samples to sliding window *W*  7:      **for all** $r_{i}\in W$ **do**  8:            Translate measurement according to estimated displacement  9:      **end for**10:      Compute averaged compensated RSSI value11:      Estimate distances to access points12:      Compute device position using multilateration13:**end while**

The sliding window *W* has a fixed length of 10 Wi-Fi samples, corresponding to approximately 20 s at the nominal Wi-Fi sampling rate of 0.5 Hz. Samples are appended in chronological order; when the window is full, the oldest sample is discarded. No explicit outlier rejection is applied; robustness to noise is achieved through the averaging operation. Parameter sensitivity analysis of the window length is identified as future work.

## 5. Experimental Setup

The system introduced in the preceding section underwent testing in controlled laboratory conditions. Due to the absence of publicly available datasets containing synchronized Wi-Fi, accelerometer, gyroscope, and magnetometer recordings alongside reference data, we conducted the measurements. To enable a meaningful comparative analysis with other established localization algorithms, we established a dedicated laboratory environment where practical experiments were executed.

A critical aspect of equitable comparisons among various localization methods and algorithms pertains to the accurate determination of a reference location. This reference location must exhibit significantly greater accuracy than that of the system under evaluation. This challenge is particularly pronounced for algorithms focusing on the localization of moving objects. In light of literature findings [50] and our experiments suggesting that Wi-Fi RSSI-based localization systems typically achieve accuracies ranging from 2 m to 10 m, we posited that the reference localization system must achieve accuracy at least an order of magnitude superior. To fulfill this requirement, a self-driving robot equipped with an optical line-following system was employed. Leveraging the sensitivity of the optical system and the width of the line, a reference localization accuracy of 2 cm was achieved, deemed sufficient for the system under examination.

Prior to each measurement session, the inertial sensors (accelerometer, gyroscope, and magnetometer) were calibrated using the optical ArUco-based subsystem. The initial device pose obtained from the ArUco marker was used to establish the reference coordinate frame and correct sensor biases and orientation offsets. Wi-Fi access point positions were surveyed manually and verified against the floor plan. No additional hardware calibration was performed.

The experimental setup involved a Xiaomi Redmi Note 10 mobile device featuring an ArUco code for localization, positioned at the initial measurement point using the optical localization system. Although the experiments were conducted using a robotic platform to ensure precise reference trajectories and repeatability, they were performed in real indoor spaces, including laboratories and a gym. As shown in Figure 8, the experiments were conducted in representative indoor environments containing typical furniture, walls, and structural elements.

**Figure 7:**
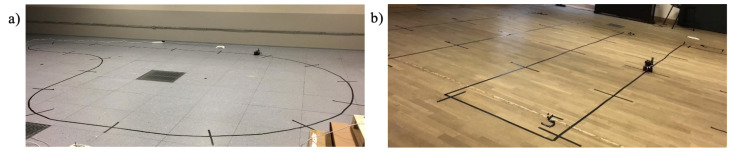
Photographs of the measurement tracks used in the experiments. Photograph (**a**) corresponds to the layout shown in Figure 8a, while photograph (**b**) corresponds to the layout shown in Figure 8b.

Figure 7, the experiments were performed in representative indoor environments containing typical furniture, walls, and structural elements. The mobile device was outfitted with the following sensors:

- A Wi-Fi network interface card, which gauges the RSSI.
- A magnetometer, which gauges the magnetic field intensity.
- A gyroscope, which measures the angular velocity of the mobile device across three axes (roll, pitch, yaw).
- An accelerometer, which measures the acceleration experienced by the mobile device.

**Figure 8:**
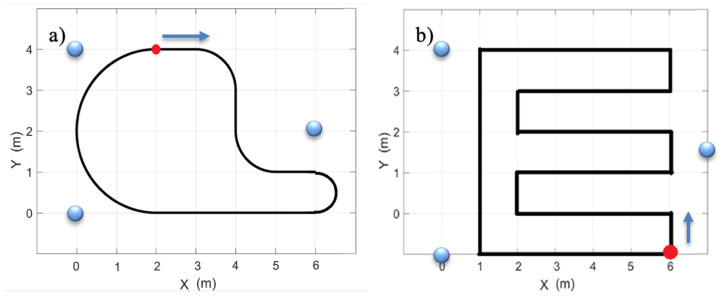
Schematic diagram of the measurement tracks used in the experiments: (**a**) track inspired by the EvAAL competition layout; (**b**) track covering the entire laboratory space. Wi-Fi access point locations are marked in blue, starting points in red, and arrows indicate the direction of movement.

The data obtained from these sensors were systematically recorded for subsequent analysis to compare the proposed localization algorithm with alternative approaches. To ensure measurement reproducibility, two distinct test tracks were constructed under consistent conditions. The first track, shown in Figure 8a, is inspired by the reference track established for the EvAAL competition, providing a standardized platform for evaluating indoor localization algorithms [18]. The second track, shown in Figure 8b, covers the entire laboratory space where the experiments were conducted.

The positions of Wi-Fi access points utilized for localization purposes are denoted by blue markers. Each trajectory involved localization relative to the coordinate frameworks depicted in Figure 8a,b. Throughout the experiment, the robot initiated its motion from a predetermined point, indicated by a red marker, where a camera paired with the ArUco localization system was affixed. Subsequently, it traversed along the delineated path. As the robot progressed, the designated sensors captured data, which was logged on the mobile device. The ArUco recorder function was fulfilled by a computer equipped with a 14 Mpix resolution camera and software leveraging the ArUco library [51]. To ensure synchronization and consistency between data collected from both systems, all measurement instances were temporally stamped, and the timing across the robot’s measurement apparatus and the ArUco measurement system was meticulously synchronized prior to each experimental run.

These tracks were evaluated in four different laboratory settings to assess the system’s performance under varying external conditions. Images of selected locations featuring the described tracks are provided. The displayed photo illustrates the track patterned after the EvAAL competition layout, while the subsequent image showcases the layout covering the entire laboratory area.

Each of the tracks was established across four distinct locations within the premises of our university, as detailed below:

- Room no. OO2B of building B19.
- Room no. 0.25 of building B15.
- Room F8 of building B9.
- A gym located in building B28.

Across each track and location, a series of experiments were conducted to capture both localization data and reference data. The reference data was acquired through an optical system integrated into the robot. As the optical system intersected lines perpendicular to the track, as illustrated in Figure 7, it documented synchronization markers within the data, facilitating the determination of the robot’s reference position at each intersection. It was assumed that the robot maintained a constant velocity between these intersections, enabling the determination of the reference location at any given moment. Research conducted on the track depicted in Figure 7a encompassed three distinct robot velocities: 0.2 m/s, 0.4 m/s, and 0.6 m/s. Conversely, due to the sharp curves within the track depicted in Figure 7b, the robot’s maximum speed was constrained to 0.2 m/s. For each conducted experiment, the system affixed to the robot captured the following data:

1. The Wi-Fi RSSI value from each of the three access points, measured by the mobile device’s wireless network card.
2. Data derived from the mobile device’s accelerometer, gyroscope, and magnetometer.
3. The initial position determined using the ArUco system.
4. The reference position determined based on the optical system.

The data collected above served as inputs for determining the robot’s location utilizing various localization methods, including the method introduced in this study.

## 6. Results and Discussion

**Figure 9:**
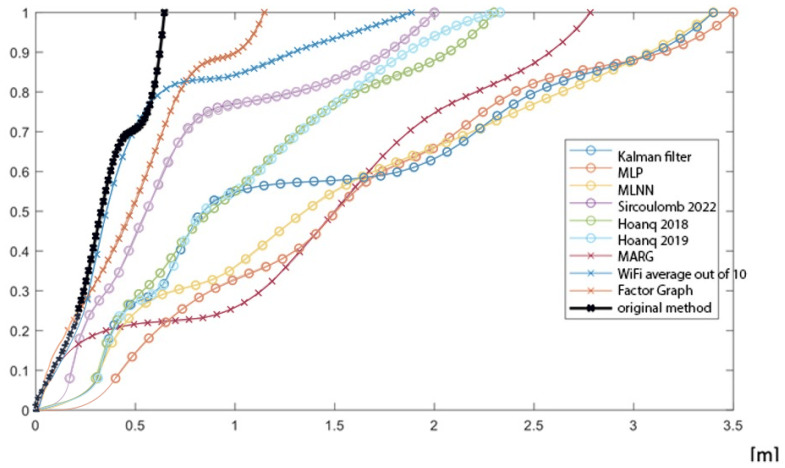
Comparison of localization results: cumulative distribution function (CDF) of the localization error for the evaluated methods [56,57,58].

In this section, we present the results of the experiments conducted using the setup described in the previous section. For an objective comparison of different localization algorithms, we use standard accuracy metrics: average error (AE), maximum error (MaxE), standard deviation (SD), mean square error (MSE), skewness (Sk), kurtosis (K), and the cumulative distribution function (CDF). The average results obtained from the four test tracks are presented in Table 3 and Figure 9.

To assess the impact of optical initialization on localization accuracy, we additionally evaluated the system without ArUco-based pose estimation. In this scenario, the initial device position was assumed to be the origin of the coordinate system with zero heading error. The results are summarized in Table 1. ArUco initialization significantly improves accuracy, particularly in terms of maximum error and standard deviation, by providing a calibrated starting point for the fusion algorithm.

For the data obtained during measurements conducted by the authors, the method utilizing a multilayer perceptron (MLP), implemented as a feedforward neural network regressing position from inertial and Wi-Fi features, as described in [52], yielded the poorest outcomes. According to the CDF depicted in Figure 9, the localization determined using this method generally contains the highest number of localization errors as well as the highest values of both mean and maximum error. Similar outcomes were obtained for the very similar multilayer neural network (MLNN) algorithm published in [53], which uses a comparable architecture but is trained with different optimization parameters and data preprocessing.

Slightly better results were achieved by the algorithm employing the Kalman filter, a classical recursive estimator based on linear state-space modeling and Gaussian noise assumptions [54]. In line with the results presented in Figure 9, the localization determined using this method entails a smaller number of errors on average, whereas the number of high-value localization errors is similar to the results obtained by the MLP and MLNN methods. Similarly to these two methods, the maximum error value is approximately 3.5 m, which practically renders these methods unusable in the considered conditions.

To provide further insight into the contribution of each system component, Table 2 presents the localization accuracy achieved by individual modules in isolation: IMU-only dead reckoning, Wi-Fi RSSI trilateration without motion compensation, and the full proposed fusion. These results confirm that each component contributes meaningfully to the final accuracy. Although the triangulation-based multilateration approach described by Equations (9)–(13) was implemented and tested, it was ultimately subsumed within the Factor Graph fusion framework, which yielded superior results. The geometric formulas are presented for completeness and to describe the distance-to-position conversion step used internally by the Factor Graph algorithm.

The Magnetic, Angular Rate, and Gravity (MARG) [55] approach, based solely on inertial sensor fusion without external signal correction, performs comparably to the Kalman filter.

Slightly better results were obtained for the Hoang algorithms [56,57] in both tested versions. These methods rely on recurrent neural networks (RNNs) to model time-series dependencies in RSSI and inertial data and estimate movement trajectories. The 2019 version incorporates improvements to model architecture and training strategy, offering slightly improved accuracy compared to the 2018 variant. Nonetheless, the maximum error value of approximately 2.3 m still is not a satisfactory outcome in the considered applications. Coupled with the observation that approximately 40% of measurements contain errors above 1 m, approximately 20% of measurements obtained using the 2018 Hoang method, and 5% of measurements obtained using the 2019 Hoang method contain errors exceeding 2 m, also prohibits the practical application of this method.

Significantly better results are achieved by the Sircoulomb 2022 method [58], which applies a constrained Kalman filter adapted to indoor localization scenarios. As despite a similar maximum error value, over 70% of localizations determined using this algorithm contain errors below 0.5 m, and 80% of measurements determined using this algorithm contain errors below 1.5 m, this method is substantially more effective under controlled conditions.

Although the Kalman filter is theoretically optimal under the assumption of linear system dynamics, more recent approaches also employ deep learning and hybrid sensor fusion techniques [13,14]. However, its application in the considered scenario is limited by several practical constraints. First, the low frequency of Wi-Fi RSSI measurements (typically one sample every 1–2 s) is insufficient to reliably track a moving object using a recursive filter alone. Second, the system is not strictly linear due to non-Gaussian noise characteristics of sensor fusion and signal propagation, and the individual measurements are not statistically independent, especially for RSSI values collected during motion. While Extended Kalman Filters (EKF) [59], Unscented Kalman Filters (UKF) [60], or Particle Filters (PF) [61] could theoretically improve performance, they would still not resolve the fundamental issue of inadequate Wi-Fi sample density in relation to the object’s motion speed.

To address this, the proposed method incorporates a compensation step using inertial sensor data. This allows previously collected Wi-Fi RSSI samples to be virtually “shifted” back to the current location estimate based on motion, enabling the use of a simple average as a statistically grounded estimator under the assumption of Gaussian-distributed measurement noise. In practice, while the accuracy of this approach depends on the precision of the inertial navigation system, experimental results confirm that the proposed method outperforms classical filtering techniques under the evaluated conditions.

Furthermore, in crowded environments where human bodies act as additional signal absorbers and reflectors, the RSSI distribution may deviate further from the Gaussian assumption, potentially degrading the performance of the proposed averaging-based compensation. This effect is acknowledged as a limitation and will be evaluated in future work.

The localization algorithm proposed by the authors, which involves solely averaging compensated values of Wi-Fi RSSI measurements, labeled as “Wi-Fi AVG” in Figure 9, even without the utilization of additional error reduction methods, allows for errors below 0.5 m for approximately 80% of the tested localizations, which is a satisfactory outcome. Unfortunately, as observed in the chart presented in Figure 9, for approximately 20% of the robot’s movement-based localizations, the error for this method escalates from 0.5 m to 2 m, which is not an acceptable value in practical applications. Another localization method depicted in Figure 9 employs the Factor Graph algorithm. This method attains the smallest maximum error values, at 1 m, whereas error values for 80% of measurement samples yield worse results compared to the method relying on averaging compensated values of Wi-Fi RSSI measurements. As a result, the authors of this paper opted to combine these two methods, assuming that the Factor Graph method would lead to a reduction in large localization error values, including the maximum error while averaging compensated values of Wi-Fi RSSI measurements would facilitate the reduction of error values for the remaining measurements. The results of merging these two methods proved better than expected, as the error values for 80% of measurements are indeed at a similar level compared to the method relying on averaging compensated values of Wi-Fi RSSI measurements alone, while the remaining 20% of localized positions exhibit lower errors than those obtained using each method separately. In the authors’ assessment, this originates from the fact that each method utilizes measurements of a heterogeneous nature. Regarding computational feasibility, the execution time of the Factor Graph-based fusion algorithm is negligible in the intended application scenario. Since the system targets human movement in indoor environments, where localization updates are required at moderate frequencies, the computational complexity of the method does not pose a bottleneck on standard mobile hardware or edge devices.

The measurement errors of both methods have distinct characteristics, whereby for measurements where one method is burdened with relatively large errors, the results of the second method compensate for these errors, resulting in the outcomes of the author’s method presented in Figure 9, which, apart from the narrow range of 0.5–0.6 m, yield the best results among all tested methods. The results presented in Figure 9 also find confirmation in the values of localization error metrics presented in Table 3, where the comparison results of remaining accuracy metrics are compiled. The proposed method achieves the best results across all evaluated metrics, confirming its effectiveness.

We are aware that the results obtained for RSSI-based distance estimation are valid under the presented measurement system and idealized conditions. In real-world deployments, the accuracy of the system is expected to be lower due to various propagation effects. Notably, fast fading caused by multipath propagation was considered in our model, but its relation to the speed of the mobile device was not explicitly analyzed. We acknowledge that when the user stops moving, signal fluctuations caused by fast fading may differ in character and potentially influence the accuracy of distance estimation. This issue will be considered in future extensions of the algorithm.

Moreover, slow fading effects are known to depend not only on distance but also on environmental factors such as the shape of the room, surface materials, and the presence of obstacles. While our experiments were conducted in a mostly empty environment (as shown in Figure 7), our system is primarily intended for use in venues such as exhibition halls, trade fairs, and large public events, where dense Wi-Fi coverage typically ensures line-of-sight (LOS) conditions to at least one access point. In such scenarios, strong LOS coverage mitigates the impact of signal obstruction, and the assumption of distance-based signal compensation remains reasonable. Nevertheless, the algorithm’s behavior under non-line-of-sight (NLOS) conditions and in highly dynamic environments will be evaluated in future work to assess its robustness. Currently, when fewer than three access points are available, it is not possible to obtain a location measurement; as a result, localization will rely solely on data from the inertial system. At the same time, our algorithm is unable to distinguish between LOS (Line-of-Sight) and NLOS (Non-Line-of-Sight) signals, which can naturally lead to localization errors resulting from this limitation. The Gaussian averaging assumption is valid primarily under LOS or near-LOS conditions. Under strong NLOS conditions, the RSSI distribution may become multimodal, rendering the sample mean a biased estimator. In such cases, robust estimators or NLOS identification preprocessing would be required. Analysis of RSSI distributions under varying occlusion conditions is identified as future work.

## 7. Limitations

The proposed method has several limitations that should be considered for practical deployment. First, the system requires at least three Wi-Fi access points with known positions. In environments with sparse Wi-Fi coverage, localization falls back to inertial-only tracking, which accumulates drift over time. Second, optical initialization via ArUco markers requires a camera with known extrinsic parameters. In edge cases where the marker is not visible, the system loses its calibrated starting point, reducing positioning accuracy. Third, the method was validated using a single smartphone model (Xiaomi Redmi Note 10). RSSI sensitivity and IMU noise characteristics vary across manufacturers, and cross-device generalizability has not yet been evaluated. Fourth, the current implementation supports 2D localization only. Extension to 3D positioning in multi-floor venues would require an additional altitude sensor, such as a barometer. Fifth, the algorithm does not distinguish between LOS and NLOS signal conditions. In highly cluttered or reflective environments, NLOS propagation may introduce systematic distance estimation errors that the current model cannot compensate. The experiments were conducted using a robotic platform, which does not capture the variability introduced by human carriers, such as body shielding of antennas, irregular gait, and changes in device orientation. Future experiments should involve human participants to provide a more realistic assessment of system performance. Finally, the filter parameters—specifically the sliding window length—were tuned to the experimental conditions (Wi-Fi at 0.5 Hz, IMU at 100 Hz, velocity below 6 km/h) and may require re-tuning for other deployment scenarios. The experimental tracks were limited in size (approx. 4 m × 6 m) and conducted predominantly under LOS conditions. Future work should include experiments in larger venues with explicit NLOS scenarios, e.g., through deliberate obstruction of signal paths or testing in multi-room environments, to better assess the generalizability of the proposed method.

## 8. Conclusions

In conclusion, the localization system proposed in this paper combines three complementary components: optical ArUco-based initialization, inertial sensor-based displacement tracking, and Wi-Fi RSSI-based positioning. The optical subsystem provides centimeter-level 6D initialization and sensor calibration at zero additional infrastructure cost. The inertial subsystem compensates for the low sampling frequency of Wi-Fi measurements by virtually translating historical RSSI samples to the current position. The combined Factor Graph fusion achieves an average localization error of approximately 0.40 m, representing a 31% improvement over the best competing method and a nearly threefold reduction in maximum error. The system operates without fingerprinting databases or specialized hardware. This makes it practical for deployment in venues such as stadiums, exhibition halls, and trade fairs where Wi-Fi infrastructure already exists. Limitations include reliance on controlled LOS conditions, single-device testing, and 2D-only localization, all of which will be addressed in future work.

The experiments conducted in this study were performed under controlled conditions with the primary objective of comparing localization algorithms using a known reference trajectory. This allowed us to precisely quantify localization errors in a reproducible manner. To ensure measurement repeatability, we employed a small-scale measurement track inspired by the EvAAL benchmarking setup. While we acknowledge that real-world environments often involve obstacles, signal obstructions, and device heterogeneity, the reproducibility and accuracy of such experiments would be significantly more difficult—if not infeasible—to achieve outside controlled laboratory settings. Additionally, we note that Wi-Fi RSS values may vary across different smartphone models, and this study was limited to a single device. These limitations are important, and addressing them will be a subject of future research. Specifically, we plan to evaluate our system in larger, obstacle-rich spaces using a variety of mobile hardware configurations to better assess its robustness and generalizability.

Future work will address cross-device validation, extension to 3D localization, and robustness in NLOS environments. Finally, the algorithm currently assumes Line-of-Sight (LOS) or near-LOS conditions typical of open exhibition halls; performance in highly cluttered, Non-Line-of-Sight (NLOS) environments requires further investigation utilizing advanced NLOS identification techniques [62]. To support reproducibility and further research, the dataset collected during the experiments, including synchronized Wi-Fi RSSI, inertial sensor data, and reference trajectories, will be publicly released.

## Figures and Tables

**Table 1:** Impact of optical (ArUco) initialization on localization accuracy. Without ArUco, the initial position was set to the coordinate system origin with zero heading.

Configuration	AE [m]	MaxE [m]	SD [m]	MSE [m^2^]
With ArUco	0.40	0.70	0.44	0.19
Without ArUco	0.54	0.99	0.61	0.34

**Table 2:** Per-module localization accuracy. IMU-only results correspond to the MARG method, which relies solely on inertial sensor fusion without external signal correction.

Module	AE [m]	MaxE [m]	SD [m]	MSE [m^2^]
IMU only (MARG)	1.61	3.00	1.83	3.35
Wi-Fi AVG only	0.68	2.05	0.84	0.71
Full system	0.40	0.70	0.44	0.19

**Table 3:** Comparison of different localization methods. AE: average error; MaxE: maximum error; SD: standard deviation; MSE: mean square error; Sk: skewness; K: kurtosis. MLP: multilayer perceptron [52]; MLNN: multilayer neural network [53]; MARG: Magnetic Angular Rate and Gravity [55]; Hoang 2018/2019 [56,57]; Sircoulomb [58].

Localization Algorithm	AE [m]	MaxE [m]	SD [m]	MSE [m^2^]	Sk [−]	K [−]
Sircoulomb	0.68	2.01	0.81	0.66	0.97	1.31
Hoang 2018	0.96	2.32	1.09	1.18	1.49	2.16
MLP	1.53	3.51	1.74	3.04	3.88	9.19
MLNN	1.57	3.41	1.87	3.50	4.84	13.5
Hoang 2019	0.97	2.33	1.09	1.19	1.48	2.11
Kalman	1.44	3.42	1.79	3.21	4.75	13.5
MARG	1.61	3.00	1.83	3.35	7.55	18.0
Wi-Fi AVG	0.68	2.05	0.84	0.71	0.99	1.60
Factor Graph	0.58	1.24	0.67	0.45	0.40	0.40
Our algorithm	0.40	0.70	0.44	0.19	0.11	0.06

## Data Availability

The dataset collected during the experiments, including synchronized Wi-Fi RSSI measurements, inertial sensor data, and reference trajectories, will be made publicly available upon publication.
